# Advancing the situational application of LEGO^®^ Serious Play^®^: a qualitative approach to exploring student transition in nursing education

**DOI:** 10.1177/17449871261489554

**Published:** 2026-11-07

**Authors:** Benjamin Olusola Ajibade

**Affiliations:** Assistant Professor of Nursing, School of Healthcare and Nursing Sciences, Faculty of Health and Wellbeing, Northumbria University, Newcastle Upon Tyne, UK; Nursing Research and Innovation, School of Healthcare and Nursing Sciences, SEETA University, Mbalala, Uganda

**Keywords:** creative methods, interpretative phenomenological analysis, LEGO^®^ Serious Play^®^, nursing education, qualitative methodology, student transition

## Abstract

**Background:**

Qualitative methods relying on verbal articulation systematically exclude participants whose experiences are shaped by power asymmetry, prior conditioning to silence and racialised environments. LEGO^®^ Serious Play^®^ (LSP) has shown potential as an inclusive approach, but its theorisation as a methodology remains underdeveloped.

**Aims:**

This paper advances a four-principle framework for the situational application of LSP as a context-responsive qualitative methodology and contribution to equity-centred nursing research.

**Methods:**

Drawing on an interpretative phenomenological analysis study with racially minoritised (RM), internationally transitioning nursing students in UK higher education, the paper examines conditions under which LSP generates access to experiences shaped by power, hierarchy and structural inequities of racialised transition.

**Results:**

Situational LSP enabled participants to materialise tacit experiences of power, identity and belonging through construction, generating spatial and symbolic data qualitatively different from verbal accounts. The framework comprises four principles: context sensitivity, participant voice and positioning, meaning-making through metaphor, and construction and researcher reflexivity.

**Conclusions:**

The situational application of LSP is a transferable methodological contribution to nursing research. For practice and education, it offers a model prioritising equity of voice among RM and transitioning nursing students. For policy it highlights the need for approaches addressing experiential complexity of internationally educated nurses navigating racialised professional transition.

## Introduction

The increasing complexity of lived experience within higher education, particularly among internationally mobile students, continues to challenge conventional qualitative research approaches. Processes such as cultural transition, identity negotiation and academic adaptation (Tinto, 1993) are often deeply embodied, emotionally layered and not easily articulated through traditional data collection methods. Within higher education contexts, student belonging, engagement and retention are closely interconnected and central to student success (Thomas, 2012), yet the experiential dimensions of these processes remain difficult to access through interview or focus group methods alone, particularly where issues of power, confidence, language and cultural positioning are at play. For racially minoritised (RM) and internationally transitioning nursing students, these challenges carry an additional structural dimension: shaped by colonial educational legacies, prior conditioning to deference and silence, and the particular power dynamics of entering a predominantly white professional sector, these students are precisely those most likely to be excluded from conventional qualitative research encounters in which verbal fluency and academic confidence function as prerequisites for participation (Ajibade and Hayes, 2022; Ajibade et al., 2026). This challenge is not merely technical; it extends to questions of epistemic justice: whose knowledge counts, and whose remains invisible within the production of academic knowledge (Moralli, 2024; Nathan et al., 2023).

Within this context, there has been a growing interest in creative, participatory and arts-based approaches to qualitative inquiry. Such methods move beyond purely discursive forms of data, recognising that meaning is often constructed through visual, symbolic and embodied processes (Leavy, 2017; Pink, 2013). These methodological developments align with broader calls for approaches that foreground co-construction, reflexivity and participant voice as central to knowledge production, and that can engage participants whose experiences may be constrained by language, hierarchy or cultural positioning (Moralli, 2024; Nathan et al., 2023). However, such methods are not inherently inclusive; their effectiveness depends critically on how they are designed, facilitated and adapted to the specific socio-cultural and relational conditions of each research encounter (Brown, 2022; Heard, 2023). This context-responsive dimension, what this paper terms situational application, has not been adequately theorised in existing methodological literature.

One such emerging approach is LEGO^®^ Serious Play^®^ (LSP), a facilitated methodology grounded in principles of constructionism, metaphorical thinking and experiential learning (Kristiansen and Rasmussen, 2014). LSP enables participants to construct three-dimensional models as representations of their experiences, used as prompts for storytelling, reflection and shared meaning-making. Although LSP has gained traction as a pedagogical and facilitation tool, its theorisation within qualitative research remains relatively underdeveloped (Henderson et al., 2025; Rose and Furness, 2024). Ajibade and Hayes (2022) utilised LSP within an interpretative phenomenological framework to explore the socio-cultural transitions of Nigerian nursing students entering United Kingdom (UK) higher education, demonstrating its capacity to facilitate deeper dialogue, reduce hierarchical barriers and enable participants to articulate complex emotional and cultural experiences through metaphor and narrative. That study constitutes the empirical foundation from which the present paper develops. The methodological approach underpinning it was first documented in Ajibade and Hayes (2020), providing the initial peer-reviewed account of LSP used in this way with transitioning students in UK higher education. More recently, Ajibade et al. (2026) directly theorised LSP’s decolonising potential, demonstrating its capacity to validate the knowledge and meaning-making of RM staff and students rather than subordinating it to dominant discursive conventions.

The current paper advances beyond the findings of that prior work in two significant ways. Firstly, although Ajibade and Hayes (2022) reported on the substantive experiences of transitioning students, the present study foregrounds the methodological dimensions of that work, interrogating how and why LSP functioned as it did within that specific research context. Secondly, drawing on deeper conceptual engagement with the qualitative methods literature, this paper articulates an explicit theoretical framework, the situational application of LSP, that was implicit but underarticulated in the earlier study. Despite the promising developments evident in the literature, much of the existing work treats LSP as a fixed method or toolkit, with limited attention to how it should be adapted in response to specific research contexts, participant groups and epistemological orientations (Henderson et al., 2025). This paper addresses that gap directly, advancing the concept of the situational application of LSP as a novel methodological contribution that repositions LSP as a contextually responsive, equity-oriented qualitative methodology and provides a four-principle framework for its principled enactment in future nursing and health research. Crucially, this methodological contribution is inseparable from its equity dimension: the framework emerged as a direct response to the structural exclusion of racially minoritised participants from research encounters that privilege verbal fluency, and its value is demonstrated precisely through that exclusion and its correction.

## Background and literature review

### Qualitative methods and the study of lived experience

Phenomenological and narrative methods have generated valuable insights into lived experience, emphasising depth, meaning and subjective interpretation (Finlay, 2011; Smith et al., 2009). However, their reliance on verbal articulation as the primary data-generation mode limits access to experiences that are embodied, emotionally complex or shaped by prior conditioning to silence (Ellis et al., 2024). This limitation is especially pronounced in research involving cross-cultural transitions, where feelings of dislocation and identity shift may not be easily captured through interview-based approaches. Traditional qualitative methods can also reproduce power dynamics within the research encounter, privileging participants who are more confident, articulate or familiar with academic discourse (Parahoo, 2014). These asymmetries are not incidental; they reflect broader structural inequities that shape whose knowledge counts and whose voice is heard within qualitative research.

### Creative and participatory approaches

Creative, participatory and arts-based approaches have emerged as a response to these limitations, demonstrating value particularly with participants whose experiences are constrained by power asymmetry, linguistic difference or prior conditioning that discourages open expression (Leavy, 2017; Moralli, 2024; Nathan et al., 2023). Such methods recognise that knowledge is not only constructed through language but also through action, imagery and material engagement (Pink, 2013). However, methodological innovation alone does not guarantee inclusivity. The effectiveness of participatory approaches depends critically on how they are designed, facilitated and adapted to the specific socio-cultural and relational conditions of each research encounter (Brown, 2022; Heard, 2023). Without this context-responsive dimension, participatory methods risk reproducing rather than redistributing the communicative hierarchies they were developed to challenge: a creative method imposed through a standardised protocol may give participants a different task, but it does not necessarily give them a different relationship to the authority of the researcher or to the institutional context in which data generation takes place. It is this reflexive, adaptive dimension, what this paper terms ‘situational application’, that the literature has not adequately theorised.

### LSP: from facilitation tool to research methodology

LSP is grounded in constructionist principles (Harel and Papert, 1991; Kristiansen and Rasmussen, 2014) and involves participants building three-dimensional models in response to open prompts, using constructions as scaffolds for storytelling, reflection and shared meaning-making. Its first peer-reviewed use with transitioning students in UK higher education was documented in Ajibade and Hayes (2020), providing the procedural and reflexive groundwork for subsequent empirical development. Ajibade and Hayes (2022) then demonstrated within an interpretive phenomenological approach (IPA) study; an approach that seeks to explore the meaning individuals make of their lived experiences, that LSP could reduce power imbalances and enable participants to articulate experiences of cultural dislocation, identity and belonging through metaphor and construction in ways that extended beyond conventional verbal accounts. More recently, Ajibade et al. (2026) theorised LSP’s decolonising potential, demonstrating its capacity to enhance the experience of racially minoritised staff and students by creating conditions in which their knowledge and ways of meaning-making are validated rather than subordinated to dominant discursive conventions. Henderson et al. (2025) positioned LSP within the broader landscape of participatory research methodologies, and Rose and Furness (2024) explored its capacity to elicit rich phenomenological data in qualitative interviews. Despite these developments, LSP is consistently treated as a technique to be applied rather than a methodology to be theorised. There remains limited engagement with how it operates epistemologically or what conditions govern whether it fulfils its emancipatory potential. This paper addresses that gap directly.

### International student transition and methodological access

Research on international student transition consistently highlights the multi-dimensionality of the experience, encompassing cultural dislocation, pedagogical adjustment, language differences and the negotiation of new academic and social identities (Birindelli, 2023; Sovic, 2008; Trahar, 2013). Recent work has demonstrated that international students occupy multiple overlapping social identities simultaneously, each contributing to a shifting sense of self across contexts (Vidal, 2025). These experiences are shaped by prior educational conditioning including norms around deference to authority, restricted participation and hierarchical classroom cultures, which may carry forward and inhibit open engagement in both educational and research settings (Hassel and Ridout, 2018). Capturing the full complexity of these experiences presents a significant methodological challenge. The reliance on verbal articulation as the primary data-generation mode may constrain access to experiences that are embodied, emotionally complex or shaped by prior conditioning to silence, situating the methodological contribution of the present paper within this specific context of need.

## Conceptual framing: the situational application of LSP

The situational application of LSP recognises that qualitative methods are not neutral tools applied uniformly across contexts but are dynamically enacted within specific social, cultural and relational environments. The theoretical foundations of the framework are developed in full in Ajibade (2026b). Critically, this framework was not developed as a response to methodological limitations in the abstract; it emerged as a direct response to the structural exclusion of racially minoritised participants from conventional research encounters in which verbal fluency and familiarity with dominant discursive conventions function as prerequisites for participation. The situational application of LSP is therefore both a methodological innovation and an equity intervention; the two dimensions are inseparable. Situational application is defined as:

the intentional adaptation of LEGO® Serious Play® in response to the specific sociocultural context, participant positioning, and epistemological aims of a research encounter, in order to facilitate deeper, more inclusive forms of meaning-making.

This framing builds on Ajibade and Hayes (2022), which demonstrated that the effectiveness of LSP resided not in the method itself but in how it was situationally enacted. The situational application framework makes this adaptive logic explicit and transferable through four interrelated principles.

### Context sensitivity

The first principle recognises that the meaning and function of LSP are inherently shaped by the context in which it is deployed. Within higher education research involving transitioning students, context includes not only institutional structures but also broader socio-cultural dynamics such as migration, race, language and prior educational experience. Situational application requires researchers to design and facilitate LSP sessions responsively rather than relying on standardised procedures.

### Participant voice and positioning

A second defining feature attends to participant positioning in relation to voice, agency and power. LSP disrupts dynamics that privilege verbal fluency by enabling participants to think with their hands, using physical construction as a precursor to articulation. Within a situational framework, however, this is not assumed as an inherent property of LSP but as an outcome that must be actively facilitated through careful session design and researcher responsiveness.

### Meaning-making through metaphor and construction

Central to LSP is the use of metaphor as a vehicle for meaning-making. Participants construct models that symbolise their experiences, which are then narrated and interpreted. This aligns with constructionist perspectives that knowledge is actively built through interaction with the world and with others (Gauntlett, 2007; Harel and Papert, 1991). Within situational application, the emphasis shifts from simply using metaphor to understanding how metaphor operates within specific contexts, foregrounding the interpretive richness of constructions as data that are both individually meaningful and socially situated.

### Researcher reflexivity and facilitation

Finally, the situational application of LSP foregrounds the researcher as an active facilitator whose prompt design, session structure and management of group dynamics all influence how data are generated. In contexts where the researcher shares cultural or experiential background with participants, reflexivity is particularly critical for managing assumptions and maintaining interpretive openness. Situational application requires a reflexive stance in which the researcher continuously engages with their own influence within the research encounter.

The four principles are illustrated schematically in Figure 1.

**Figure 1.**
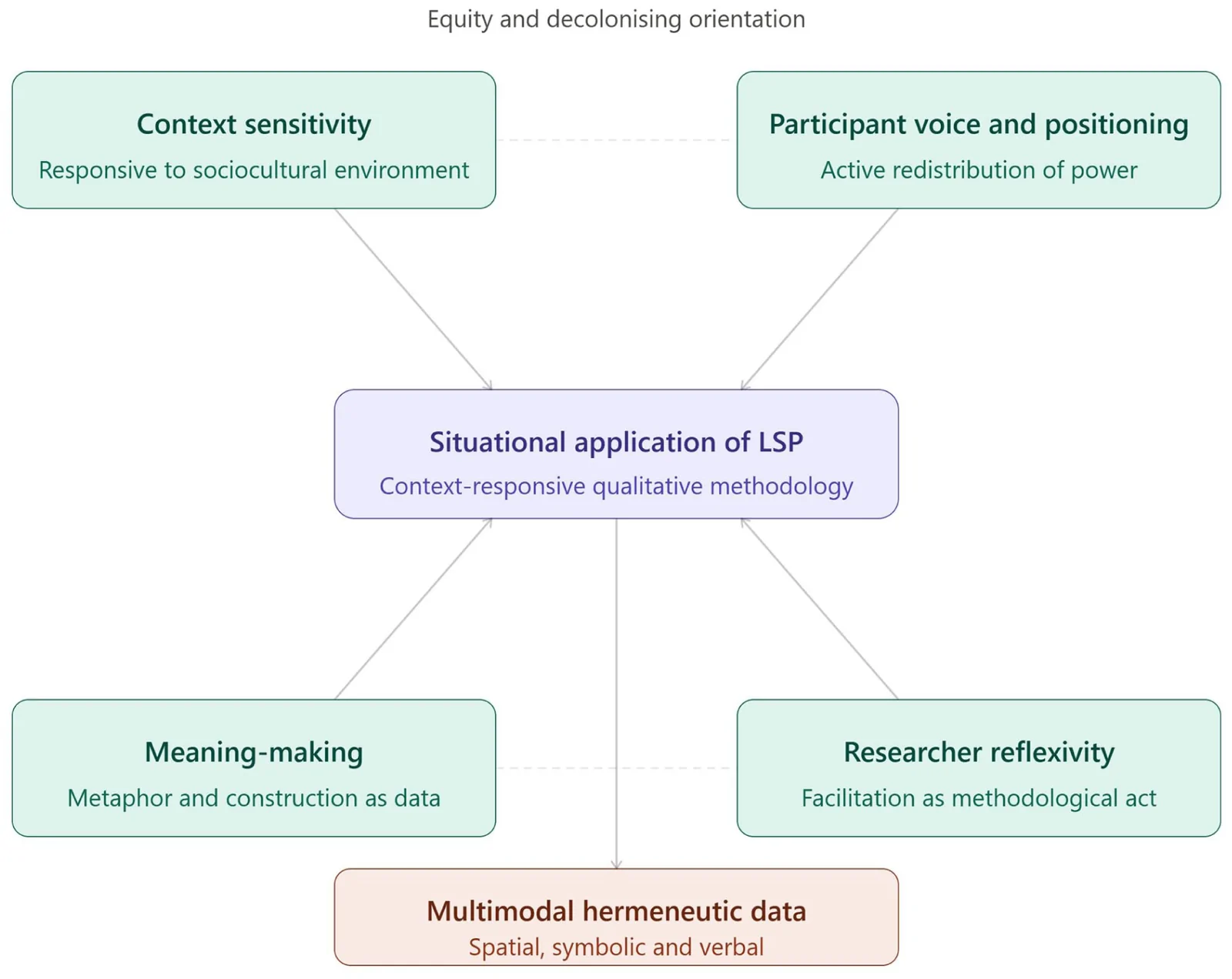
The situational application of LSP framework.

## Methodology

### Research design

This study adopts a qualitative design grounded in Interpretive Phenomenological Analysis (IPA), with LSP employed as a situationally adapted data collection method. IPA is suited to studies of transition where experiences are complex, subjective and deeply embedded in socio-cultural context (Finlay, 2011; Smith et al., 2009). The integration of LSP within IPA reflects a methodological alignment between phenomenological inquiry and constructionist principles: while IPA seeks to access lived experience through in-depth exploration of personal meaning, LSP enables participants to externalise those experiences through metaphorical construction, creating an additional mode of expression that extends beyond the verbal. The methodological approach has evolved across three published works: Ajibade and Hayes (2020) documented the procedural and reflexive dimensions; Ajibade and Hayes (2022) reported the substantive empirical findings and the present paper subjects that combined approach to systematic theoretical scrutiny, articulating the situational application framework that was implicit but unarticulated across both earlier studies. To be explicit about the nature of this contribution: the present paper is a conceptual and methodological re-theorisation drawing on previously published empirical work. It does not constitute re-analysis of raw data or a new empirical study, but rather a theoretically informed reconstruction of the methodological logic that underpinned the original inquiry, developed through sustained engagement with the qualitative methods literature and accumulated reflexive insight into how and why LSP functioned as it did within that specific research context. This form of contribution, the retrospective articulation of a framework implicit in prior empirical work, is a recognised and legitimate mode of methodological scholarship (Finlay, 2011; Smith et al., 2009).

### Participants and sampling

A purposive sampling strategy was employed to recruit participants with direct lived experience of transitioning into UK higher education as internationally mobile students, consistent with IPA principles for recruiting those best placed to provide rich, in-depth insights into the phenomenon under investigation (Shorten and Moorley, 2014; Smith et al., 2009). Twenty participants (*n* = 20) were recruited from an annual cohort of Nigerian nursing students at a satellite campus of a Higher Education Institution in the north of England. All were undergraduate nursing students nearing completion of, or who had recently completed, their programmes. Small group sessions of four to six participants facilitated interaction while maintaining a safe and supportive environment for sharing personal experiences, ensuring no single voice dominated proceedings.

### Data collection

Data were generated through facilitated LSP workshops structured around posing the question, construction, sharing and reflection (Kristiansen and Rasmussen, 2014). In line with the situational application framework, these processes were enacted responsively rather than applied in a standardised manner. Prompts were carefully developed to elicit deep reflection on key dimensions of transition and were intentionally open-ended, allowing participants to interpret and respond in personally and culturally meaningful ways. Examples include: ‘Build a model that represents your experience of learning before you came to the UK’; ‘Construct something that shows the challenges you have faced since arriving’ and ‘Build a model that represents how you see yourself now as a student in this environment’. The sequencing moved participants progressively from past experience to present reflection, creating a temporal narrative arc aligned with IPA’s interest in how individuals make sense of experience over time. Facilitation was guided by principles of inclusivity and equality of voice. Each participant built an individual model before sharing, ensuring personal perspectives were developed prior to group discussion. Data were generated through audio recordings of participant narratives, photographic documentation of models and facilitator field notes, capturing both verbal and non-verbal dimensions of experience.

### Data analysis

Analysis followed the six-stage IPA process (Smith, 2010): immersive reading and re-reading; initial noting; development of emergent themes within individual cases; identification of connections across themes; cross-case analysis and articulation of overarching themes representative of the dataset. This process was extended to incorporate what this paper terms a multimodal hermeneutic: a systematic analytical engagement with non-verbal data generated through LSP, conducted alongside rather than separately from the analysis of participant verbal narratives. Specifically, photographs of each completed model were reviewed in conjunction with audio-recorded transcripts, with analysis attending to three distinct dimensions of the material data: the spatial relationships between elements within each model (proximity, separation, scale and orientation of figures and structures); the symbolic and metaphorical choices embedded in participants’ building decisions (colour selection, structural form, the use of figures to represent self and others) and the temporal relationship between construction and narration, specifically what was built before any verbal account was offered and how the spoken narrative subsequently elaborated, modified or departed from the model’s visual logic. Participant-generated meanings were distinguished from researcher interpretations through a deliberate hierarchical principle: the participant’s own verbal account of what their model represented was treated as the primary source of meaning, and researcher observations about spatial or symbolic elements were treated as second-order analytical commentary, constrained by and tested against the participant’s stated account. Where a researcher’s reading of a model diverged from the participant’s own account, the participant’s account took precedence. This reflects both IPA’s emphasis on participant sense-making as the primary interpretive object, and the situational application framework’s principle of researcher reflexivity, which requires continuous scrutiny of the researcher’s own interpretive influence. This multimodal hermeneutic extends IPA’s double hermeneutic into new territory: the researcher makes sense not only of what participants say about their experience, but of what they have built, and of how the material and verbal dimensions of the account relate to each other. The concept of the multimodal hermeneutic is identified here as a distinct theoretical contribution that warrants further development within IPA methodology. Trustworthiness was maintained through prolonged engagement, peer debriefing, member checking and a transparent audit trail (Tracy, 2010; Yardley, 2000).

### Researcher positionality

The first author’s shared cultural background with participants offered advantages in relational rapport and cultural sensitivity while requiring careful reflexive management to avoid inadvertent imposition of assumptions or closure of interpretive possibilities. Reflexivity was practised throughout via a research journal, regular supervision discussions and member checking, consistent with established IPA conventions (Smith et al., 2009).

### Ethical considerations

Ethical approval was granted by the University of Sunderland Ethics Committee (Reference: 001745). All participants provided informed written consent prior to involvement, having received a full participant information sheet detailing the study’s nature, purpose and the ways in which data would be used and stored. Participants were explicitly informed of their right to withdraw at any point and without requirement to provide a reason, and this right was verbally reiterated at the opening of each session (Smith et al., 2009). Confidentiality was maintained throughout: all participant names in this paper are pseudonyms, no identifying institutional details are included in any published account and audio recordings were stored securely and accessible only to the research team. The use of LEGO model photographs as research data was addressed explicitly within the consent process: participants were informed, separately and in addition to general consent, that photographs of their constructions would be taken, retained as research data and potentially included in publications, with their verbal consent confirmed before any photography took place. Participants were also informed of their right not to have their model photographed without affecting continued participation in the study. Given the sensitive nature of the subject matter, including participants’ experiences of fear, marginalisation, cultural dislocation and identity negotiation, particular attention was given to psychological safety within each session: sufficient time was allocated at the opening of each session to establish ground rules and relational rapport before any building began (Ajibade and Hayes, 2022), and debriefing was offered following each workshop. Facilitators remained attentive to signs of distress throughout, and clear signposting to student support services was available if required.

## Findings: situational application in practice

Findings are organised around three core dimensions of lived experience: fear and power; transition and identity and voice and participation. Each subsection is used explicitly to illuminate the methodological principles of situational LSP that generated or shaped the data. The findings are therefore presented not only as substantive insights into student transition but also as evidence of what situational LSP does differently as a methodology. Participant names are pseudonyms.

### Constructing fear and power: materialising what language cannot hold

The context sensitivity principle holds that LSP must be designed and facilitated responsively to the socio-cultural environment of participants, including the power structures and prior educational conditioning they bring into the research encounter. Participants frequently used colour, spatial separation and structural barriers within their models to represent relationships between themselves and educators. The construction process functioned as material thinking through which experience was organised, externalised and made narratable before participants found language for it. Obianuju’s model used red bricks as barriers between herself and a figure representing her lecturer: ‘*This is my lecturers on one side and this is me. . . you can see that’s a demarcation. . . that red there is a challenge which I myself have to cross*’ (Obianuju, see Figure 2).

**Figure 2.**
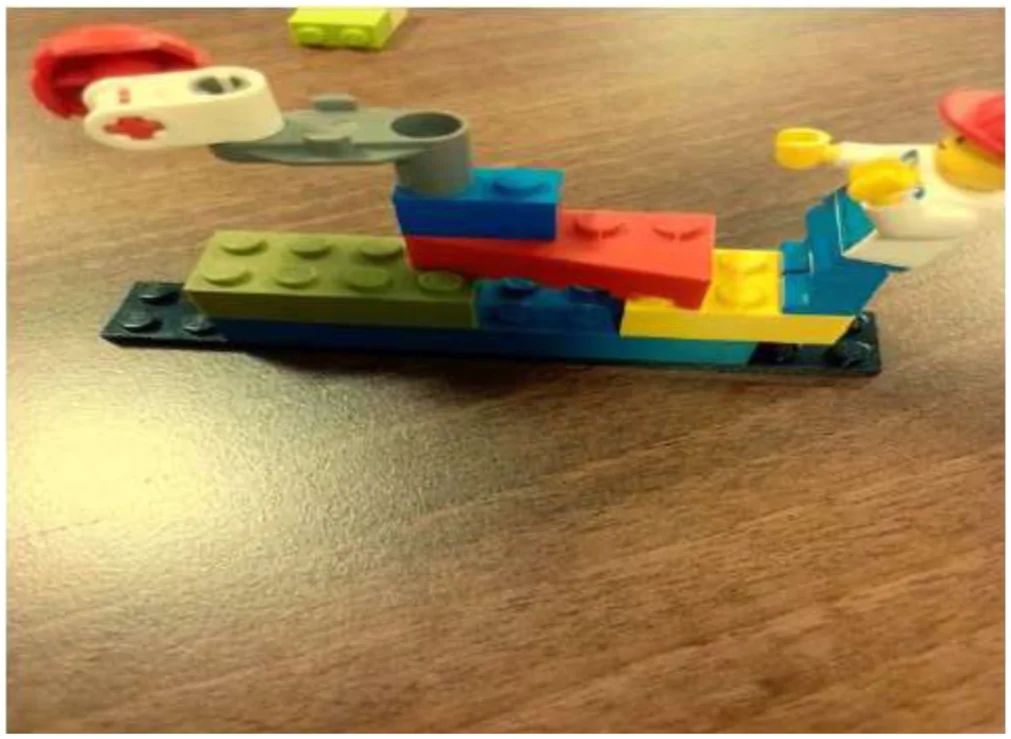
Obianuju’s LEGO^®^ Serious Play^®^ model depicting the power relationship between student and lecturer. Red bricks represent the barrier she describes as a demarcation, a challenge she must cross. The spatial separation of figures materialises a hierarchical distance shaped by prior educational conditioning that proved difficult to articulate verbally before construction.

The methodological significance lies not simply in the content but in how it became expressible. Obianuju did not begin with a verbal account of power dynamics; she built one. The model made the power imbalance visible and spatially concrete before it was narrated. This materialisation, the capacity of LSP to transform tacit experience into spatial and symbolic representation, generates forms of data not readily accessible through verbal methods alone. Participant testimony directly addresses this: ‘*I would not think of going to this in-depth without the use of LSP*’ (Alima, see Figure 3).

**Figure 3.**
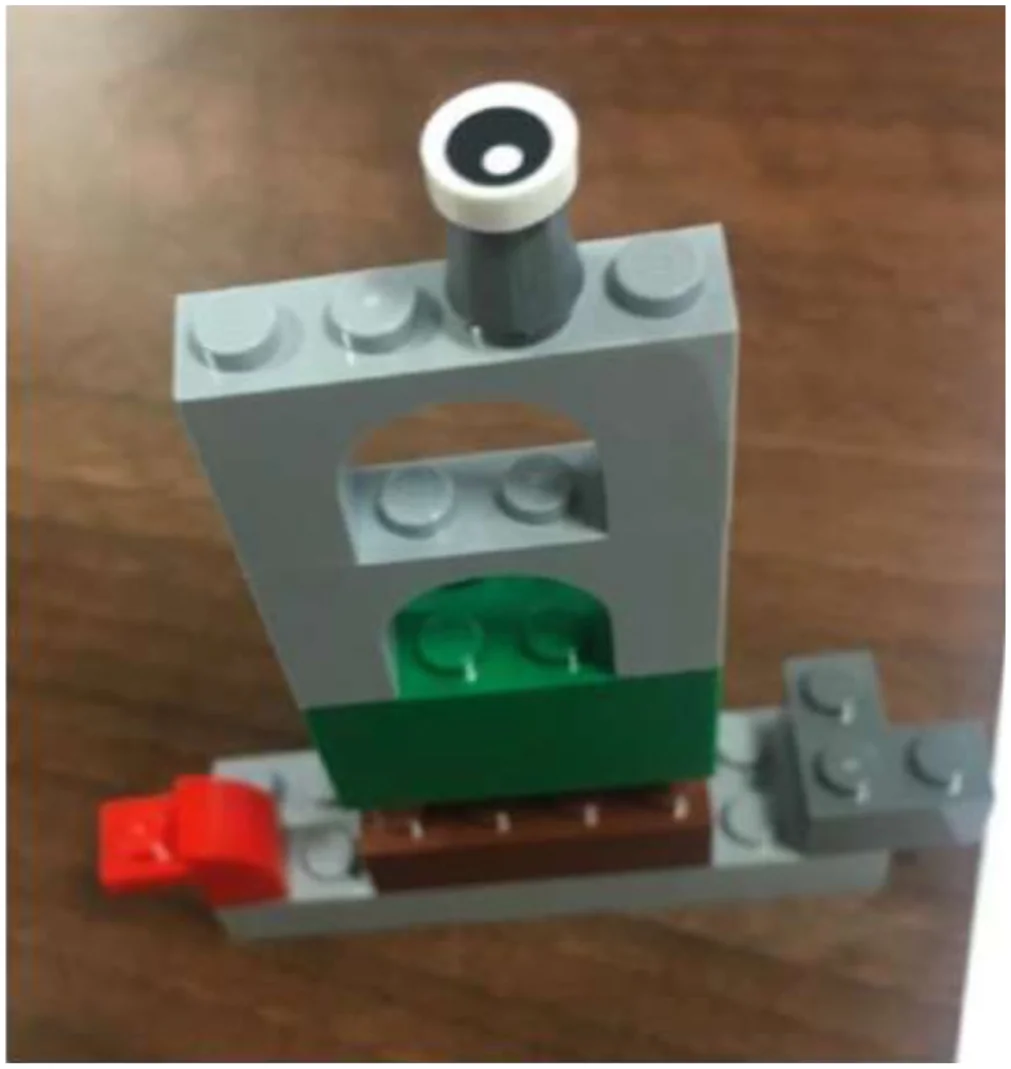
Olivia’s LEGO^®^ Serious Play^®^ model representing her experience of transition between Nigerian and UK higher education contexts. Two figures positioned at either end of a bridge, constructed in contrasting colours, represent the self she was before leaving Nigeria and the self she has become — held simultaneously within a single construction rather than resolved into a linear narrative.

This is not a general endorsement of LSP; it is evidence that situational application, the particular way the method was adapted and facilitated in this specific research encounter, enabled a form of meaning-making the participant believes would not have occurred otherwise. This constitutes the evidential core of the situational application argument.

### Transition, identity and simultaneous contradiction

The meaning-making through metaphor and construction principle holds that LSP enables participants to represent multiple, simultaneous and sometimes contradictory aspects of experience within a single physical construct. Participants used pathways, bridges, contrasting structures and figures positioned between two spaces to represent movement between educational and cultural contexts. Crucially, many participants held challenge and growth, dislocation and aspiration, belonging and exclusion, within the same model simultaneously. Olivia’s construction depicted two figures at different stages of a journey separated by a bridge, with contrasting colours representing her sense of self before and after transition: ‘*The Olivia that left Nigeria. . . is not the same person now because I see myself in a new light*’ (Olivia).

In a conventional interview, this statement might be elicited through direct questioning. What would be less accessible is the spatial relationship between the two representations, the deliberate construction of the bridge, and the fact that the participant physically arranged these elements before speaking. The construction process preceded and shaped the verbal account. This illustrates what constructionist theory predicts: meaning-making through construction generates forms of understanding qualitatively different from those produced through purely verbal methods (Harel and Papert, 1991). The situational design of prompts was central: asking participants to build their transition rather than describe it gave permission to represent complexity rather than resolve it, a methodological condition that verbal methods cannot replicate.

### Voice, silence and the redistribution of communicative power

The participant voice and positioning and researcher reflexivity principles converge most clearly in findings concerning how LSP enabled participants conditioned to silence to become active and substantive contributors. The structured architecture of the LSP workshop addressed prior communicative conditioning directly: because each participant was required to build and then share their individual model before any group discussion, participation was embedded in the design rather than left to individual confidence or verbal fluency. Gladys’s account captures this: ‘*I was able to communicate my feeling, express myself in the best possible way – talk to people – air my views – so I like that opportunity given to me*’ (Gladys).

The phrase ‘opportunity given to me’ is methodologically significant: it suggests participation was actively created by the session design, not merely available. Jane’s reflection elaborates on the relationship between the method and access to experience: ‘*I was surprised that I could say what had been kept in my mind for a very long time because I see [myself] as an introvert who will rather not say many things out*’ (Jane).

Jane identifies LSP as having enabled access to experiences that had not previously found a form, directly naming the communicative barrier that situational LSP was designed to address. Her self-identification as an introvert who would ‘*rather not say many things out*’ names precisely the participant whose voice is structurally excluded from conventional qualitative research encounters. The researcher’s reflexive management, including shared cultural background and deliberate attention to pacing, tone and psychological safety, was critical in creating conditions for such expression.

## Discussion

The findings demonstrate how the situational application of LSP generated access to dimensions of experience that extend beyond what conventional qualitative approaches typically reach. At the substantive level, they illuminate transition as emotionally embedded, identity-constituting and structurally shaped by prior educational cultures. At the methodological level, they demonstrate how situational LSP created conditions, material, spatial, relational and reflexive, that are qualitatively different from those of conventional methods.

### Beyond verbal articulation: what situational LSP adds to phenomenological inquiry

The study provides direct empirical evidence that situational LSP addresses the epistemological limitations of verbal-only approaches in practice. Participants did not simply describe their experiences once invited; the construction process preceded and shaped their verbal accounts, functioning as material thinking through which experience was organised, externalised and made narratable. This is consistent with constructionist theory (Harel and Papert, 1991) and with wider arts-based methodologies literature (Henderson et al., 2025; Leavy, 2017). The situational application of LSP extends IPA’s double hermeneutic into a new register: the researcher makes sense not only of what participants say but also of what they have built, of the spatial and symbolic decisions embedded in each model and of the relationship between material construction and verbal narration. This multi-modal hermeneutic deepens rather than departs from IPA, opening a wider aperture onto the tacit and embodied dimensions of lived experience that IPA has always sought, but that verbal-only methods can only partially access.

### Power, participation and the architecture of voice

A central contribution lies in the theorisation of how situational LSP restructures communicative power within the research encounter. Two specific mechanisms are identified. The first is structural: the design of the LSP workshop creates an equitable opportunity for contribution by sequencing each participant’s individual building and sharing before any collective discussion begins. It is important to be precise about what this achieves and what it does not. It does not remove participants’ right to remain silent or to decline to share; an equity-centred methodology must preserve the legitimacy of silence as a participant choice, and participants retained full autonomy over what they chose to disclose throughout. What the architectural sequencing removes is the structural disadvantage that faces participants for whom spontaneous verbal contribution is more costly because of prior conditioning, cultural positioning or introversion, than it is for others. By ensuring that every participant has already built before any sharing begins, the design shifts the default from speaking up to speaking from; from a situation in which contribution requires active self-assertion, to one in which it follows naturally from a model already in hand. The second mechanism is material: the act of constructing a physical model prior to speaking changes the relationship between experience and articulation in ways that reduce the authority of verbal fluency as a prerequisite for contributing something of value. Participants who described themselves as introverted, or who had been shaped by prior hierarchical educational cultures to withhold opinion, were able to contribute substantively through a process that did not require them to find words for an experience before giving it physical form. Together these mechanisms create communicative conditions that participant responses confirm were experientially significant. Critically, the findings confirm these mechanisms are not properties of LSP per se but outcomes of its situational enactment: facilitation, prompt design, pacing and active management of psychological safety were all determinants of whether LSP’s democratic potential was realised.

### Transition as simultaneous, layered and non-linear

The substantive findings advance the literature on international student transition in ways that are themselves methodologically instructive. Previous research has characterised transition as involving cultural dislocation, academic adjustment and identity negotiation (Trahar, 2013; Sovic, 2008), and recent work has demonstrated that international students occupy multiple overlapping social identities simultaneously (Vidal, 2025). The present study both confirms and complicates these accounts: the LSP data reveal that transition is not experienced as a sequential process of adjustment but as a simultaneous co-presence of contradictory states, with participants expressing dislocation and aspiration, constraint and growth, held together within a single physical construction. Verbal narrative tends towards sequence; a built model can hold contradiction without resolving it. This distinction is theorised at length in Ajibade (2026b), drawing on Bruner’s distinction between paradigmatic and narrative modes of thought: the paradigmatic mode organises experience into logical categories, whereas narrative organises it into sequence and story. A built model may occupy a third position freed from narrative’s temporal ordering because a physical object does not have to unfold in time the way a spoken sentence does and can therefore hold dislocation and aspiration, constraint and growth, as simultaneous spatial presences rather than resolved stages. This challenges more linear conceptualisations of transition (Thom, 2011) not only on substantive grounds but also on methodological ones: research instruments that tend towards linearity may systematically underreport the simultaneity and complexity of lived transition.

### Contribution to decolonising research methodology

It is important to acknowledge that LSP is a commercially produced, proprietary product originating within Western corporate and organisational development contexts, and its use is not inherently decolonising. The decolonising contribution of the situational application framework resides not in the product itself but in how situational facilitation redistributes communicative and epistemic authority within the research encounter: the creation of conditions in which participants’ own metaphors, symbolic systems and ways of organising meaning become the primary vehicles for knowledge production, rather than being subordinated to the discursive conventions of Western academic research. With this critical foundation established, the situational application of LSP aligns with and advances efforts to decolonise research practice by creating these specific conditions: conditions in which racially minoritised voices can be expressed and validated without the constraints of hierarchically dominant research designs (Ajibade, 2026b; Ajibade et al., 2026). By foregrounding participants’ own metaphors, constructions and symbolic systems as the primary vehicles for meaning-making, the framework constitutes an epistemological reorientation towards forms of knowledge that are culturally specific, embodied and non-propositional. This expands the forms through which legitimate knowledge can be generated, expressed and recognised within nursing research, contributing to the broader project of ensuring that racially minoritised and internationally mobile participants are not merely included in research but genuinely heard within it.

### Implications

Nursing as a profession carries particular hierarchical structures that shape both clinical education and the experience of internationally educated nurses entering the UK workforce. The clinical learning environment is characterised by supervisor-student power dynamics, professional gatekeeping and a predominantly white workforce receiving internationally mobile nurses, creating conditions in which the communicative barriers documented in this study are not confined to the classroom but extend into clinical practice (Ajibade, 2026a). The professional formation of a nurse asks students to develop both academic identity and clinical authority simultaneously, within a culture that historically rewards deference over challenge. For students whose prior educational conditioning has taught them that deference is the price of safety, this double demand is particularly acute. The findings reinforce the importance of designing research and educational environments that engage with this full experiential complexity. Participants’ accounts of fear instilled in Nigeria, of identity reconfiguration in the UK and of the gradual development of voice through the LSP sessions, are not incidental to academic and professional progress; they are constitutive of the conditions under which both learning and safe clinical practice become possible. The structured, inclusive architecture of situational LSP offers a pedagogical and research model for designing activities that prioritise equity of voice, consistent with established evidence on the relationship between belonging and student retention in nursing programmes (Levett-Jones and Lathlean, 2008; Levett-Jones et al., 2009). For health and social care policy, the findings underscore the need for methodological approaches capable of reaching the experiential complexity of internationally educated nurses navigating racialised professional transition. This is not a marginal concern: the UK healthcare workforce is substantially reliant on internationally educated nurses, and research that cannot access the experiential dimensions of that transition cannot inform the support, retention and integration strategies the workforce urgently requires.

### Strengths and limitations

Several limitations of this study warrant explicit acknowledgement alongside its contributions. The empirical base is situated within a specific institutional context and student population. Although the situational application framework is designed for transferability, empirical claims made here are grounded in and limited by this context, and researchers applying the framework in other settings will need to adapt it accordingly. The facilitator’s shared cultural background with participants, while a methodological strength in generating relational rapport, also constitutes a potential source of influence on what participants felt able to build and share; the data generated may not be fully separable from the specific relational dynamics of this particular research encounter. Researcher interpretation of material data carries inherent risk: the meanings attributed to spatial relationships, colour choices and structural metaphors within participants’ models are shaped by the analyst’s interpretive framework, and different researchers might read the same constructions differently. The participant-led interpretation principle described in the data analysis section above mitigates but does not eliminate this risk. Novelty effects cannot be excluded; participants encountering LEGO^®^ as a research instrument for the first time may have responded to the novelty of the method as much as to the prompts it presented, and longitudinal study would be required to determine whether the richness of data generated is stable across repeated sessions or specific to first encounters. Cultural perceptions of play as childlike or frivolous may affect engagement in some contexts, particularly where academic seriousness is valued as a marker of professional identity, as it is within some Nigerian educational cultures; this potential barrier was managed through careful framing in this study but may present differently in other settings. Physical accessibility and dexterity are not neutral variables: LSP assumes a level of fine motor engagement that may not be equally available to all participants, and this represents an equity consideration that the framework does not yet fully address. The branded and commercial nature of LSP raises questions about dependency on a proprietary product whose cost may limit accessibility for under-resourced research contexts, and whose development is controlled by a corporation rather than by the research community. This connects directly to the critical acknowledgement in the Contribution to Decolonising Research Methodology section about the product’s Western corporate origins. The study’s primary strength lies in its demonstration that situational application, rather than the method per se, is the site of methodological value: a finding that is transferable, theoretically grounded and empirically evidenced through participant testimony. Future research could extend the framework across nursing and health contexts involving refugees, migrants, and other marginalised groups; develop longitudinal applications tracking how constructions evolve over time and design comparative studies testing the methodological distinctiveness of LSP directly against verbal-only approaches with the same participants, providing a more systematic empirical basis for the claims about differential access advanced in this paper.

## Conclusion

This paper has advanced the situational application of LSP as a context-responsive qualitative methodology for nursing research. By moving beyond LSP as a facilitation tool, the study demonstrates its potential to generate rich, multi-layered insights into lived experience that extend beyond what conventional verbal methods typically reach. The four-principle framework, context sensitivity, participant voice and positioning, meaning-making through metaphor and construction and researcher reflexivity, positions LSP as an adaptable, theoretically grounded qualitative methodology that is simultaneously a methods contribution and an equity intervention.

The paper contributes to methodological debates concerning inclusion, participation and the representation of complex lived experience within nursing research; extends the epistemological foundations of phenomenological inquiry into multi-modal territory and opens productive questions about rigour, the limits of language and the decolonisation of research practice. For nursing theory, it provides a transferable framework for context-responsive qualitative methodology. For practice and policy, it offers both a research tool for understanding what belonging means to individual students and a model for creating the conditions in which equity of voice and by extension meaningful participation in both research and learning, can be genuinely realised.

**Key points for policy, practice and research**

- The situational application of LSP constitutes a rigorous, context-responsive qualitative methodology capable of generating access to experiences shaped by racialised transition and prior conditioning to silence that conventional verbal methods may not reliably reach.
- A four-principle framework, context sensitivity, participant voice and positioning, meaning-making through metaphor and construction and researcher reflexivity, provides nursing researchers with a transferable approach for designing and enacting LSP in ways that are methodologically rigorous and equity-oriented.
- The framework has direct implications for nursing education: the structured, inclusive architecture of situational LSP offers a model for designing learning environments that prioritise equity of voice and belonging among racially minoritised and internationally transitioning nursing students.
- For health and social care policy, the findings underscore the need for methodological approaches that can reach the experiential complexity of internationally educated nurses navigating racialised professional transition, knowledge essential for effective support, retention and workforce integration.
- Future research should extend the situational application framework across nursing and health research contexts and develop comparative studies that directly test the methodological distinctiveness of LSP against parallel verbal-only approaches with the same participants.

## Data Availability

The data that support the findings of this study are not publicly available due to the sensitive nature of the material and the ethical obligation to protect participant confidentiality. Data are available from the corresponding author upon reasonable request and subject to appropriate ethical and institutional approvals.
